# First person – Michelle Urman

**DOI:** 10.1242/bio.060294

**Published:** 2024-01-15

## Abstract

First Person is a series of interviews with the first authors of a selection of papers published in Biology Open, helping researchers promote themselves alongside their papers. Michelle Urman is first author on ‘ Aging disrupts spatiotemporal regulation of germline stem cells and niche integrity’, published in BiO. Michelle is a PhD student in the lab of Dr ChangHwan Lee at University at Albany, New York, investigating cellular/molecular biology focusing on cell–cell signaling.


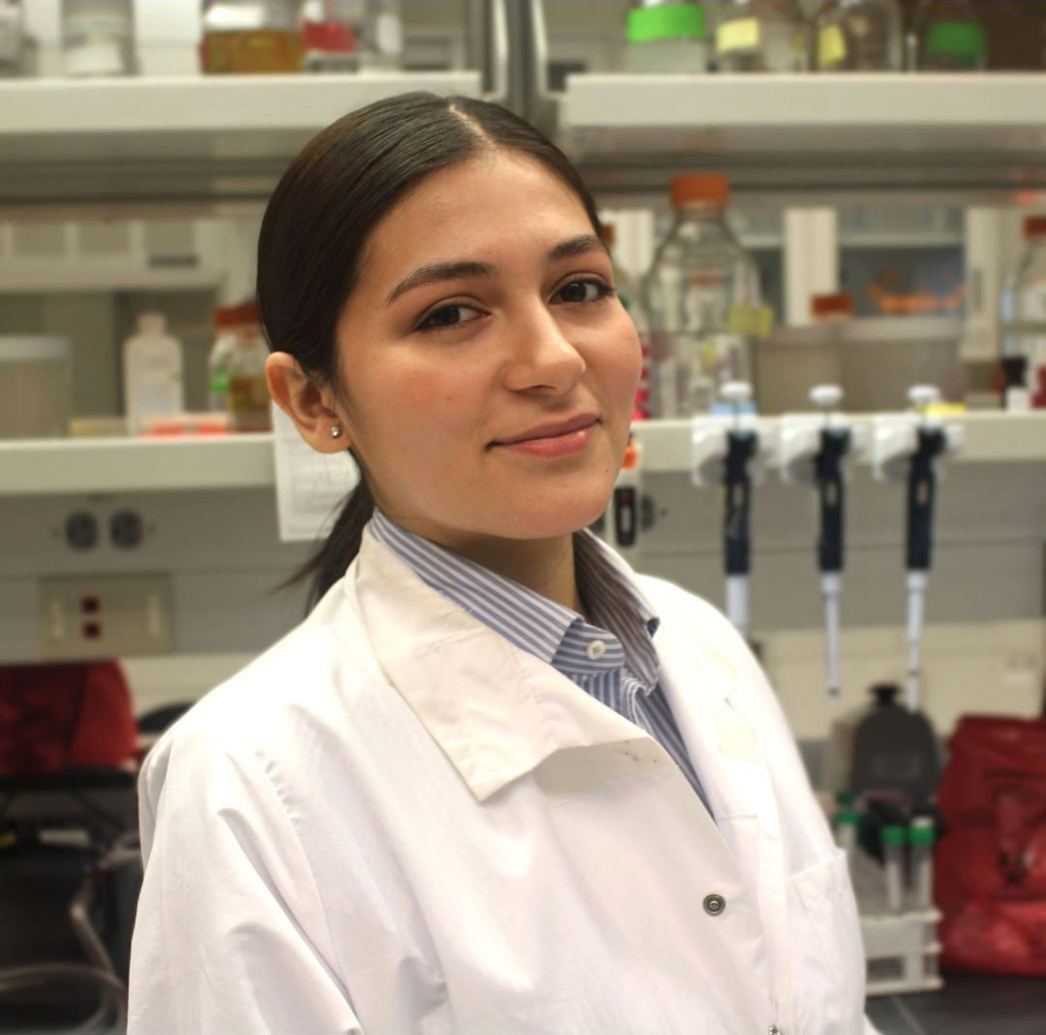


**Michelle Urman**

**Describe your scientific journey and your current research focus**

I was raised around family members that had a passion for science or medicine that had to be put aside to earn a living after immigrating to the USA. As the first child born in the USA, I had the opportunity to follow my passion and was given textbooks and a microscope as a kid to learn and observe on my own. I started my undergraduate studies as a pre-med student, but quickly changed as I was interested more in the why and how questions of what causes the disease that can lead to therapeutics.

**Who or what inspired you to become a scientist?**

My undergraduate PI, Dr Yulia Artemenko, encouraged me to apply for graduate school for a PhD and greatly inspired me to become a scientist after seeing her leadership abilities running a lab, teaching, and maintaining a strong work-life balance.

**How would you explain the main finding of your paper?**

The main finding of the paper is that the niche or microenvironment responsible for maintaining stem cells undergoes morphological changes that affect its ability to properly regulate stem cells via Notch signaling.

**What are the potential implications of this finding for your field of research?**

These findings can lead to a deeper understanding of the mechanisms of stem cell aging as well as show the use of the *C. elegans* germline as a stem cell aging model.

**Figure:**
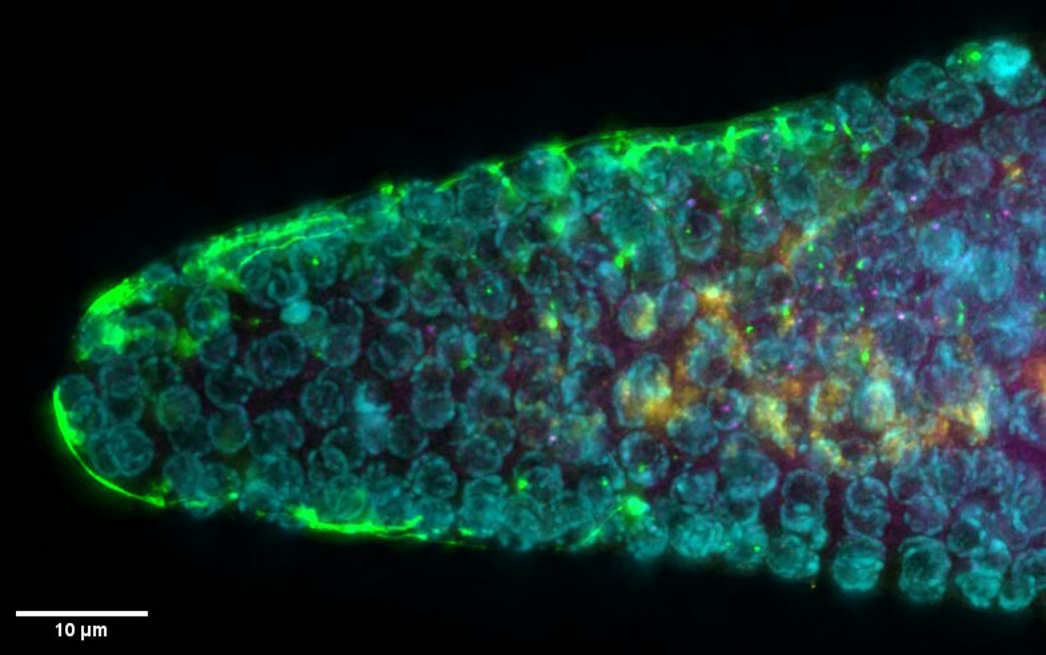
**Four-day-aged *C. elegans* germline joint smFISH and immunofluorescent staining.** Notch target gene nascent transcripts (magenta), mature mRNAs (yellow), nuclei (cyan), DTC/stem cell niche (green) simultaneously visualized.

**Which part of this research project was the most rewarding?**

The most rewarding aspect of this project was that it began as a simple rotation project during my first year of the PhD, but I was able to find some very interesting results that turned it into my first publication. I also find imaging to be a very rewarding technique as you can see the science you are studying.

**What do you enjoy most about being an early-career researcher?**

There is so much room to grow when you just start and so many options of what direction your research can go in.

**What piece of advice would you give to the next generation of researchers?**

Don't be afraid of being wrong! Instead use it as an opportunity to learn and improve yourself.

**What's next for you?**

Hopefully many more papers, a defense, and one day run my own lab!
